# Computational Modelling of Materials for Wind Turbine Blades: Selected DTU Wind Energy Activities

**DOI:** 10.3390/ma10111278

**Published:** 2017-11-08

**Authors:** Lars Pilgaard Mikkelsen, Leon Mishnaevsky

**Affiliations:** Department of Wind Energy, Technical University of Denmark, DK-4000 Roskilde, Denmark

**Keywords:** composite materials, wind energy, modeling, micromechanics, finite elements

## Abstract

Computational and analytical studies of degradation of wind turbine blade materials at the macro-, micro-, and nanoscale carried out by the modelling team of the Section Composites and Materials Mechanics, Department of Wind Energy, DTU, are reviewed. Examples of the analysis of the microstructural effects on the strength and fatigue life of composites are shown. Computational studies of degradation mechanisms of wind blade composites under tensile and compressive loading are presented. The effect of hybrid and nanoengineered structures on the performance of the composite was studied in computational experiments as well.

## 1. Introduction

The perspectives of utilization and expansion of wind energy technology depend on the reliability and lifetime of wind turbines. Degradation processes in wind turbine blades are controlled by microscale processes in the materials [1]. The mechanical behavior and strength of materials can be studied as a function of their microstructures using numerical models based on the computational micro- and mesomechanics of the materials. In this paper, methods and approaches of computational analysis of wind blade composites are discussed. The main objectives of these studies are:

○ Understanding the degradation mechanisms of wind blades as a function of their structures, and the prediction of the lifetime and performance of wind blades. For this, we estimate the loads on the wind blades (Section 2), and develop the micromechanical models of wind blade composite degradation at the level of fibers and bundles under tensile (Section 3) and compressive (Section 4) loading.
○ Exploring the promising ways to optimize the wind blade performance by tailoring the materials’ structures. Here, we develop computational models of hybrid and nanoengineered composites (Section 5) and estimate the effect of these structures on the composite performance and lifetime.

Mainly, the works carried out at the Modelling team of the Section Composites and Materials Mechanics, Department of Wind Energy, DTU, are summarized.

## 2. Loads on the Wind Blades and Stresses in the Material

In this section, a simple method to estimate loads on the wind blades is presented. There exist a number of studies estimating the loads on wind blades from aerodynamic, physical loads [2,3,4,5]. However, in this work, we seek to develop a simple, beam model-based approach, which can be introduced in the complex materials model as a boundary condition.

### 2.1. Loads on a Wind Blade

A wind turbine blade is a long, slender structure where the dominating loads are given by the aerodynamics and the gravitation. Seen on the large scale, a wind turbine blade is a simple beam structure with well-defined load and boundary conditions. The beam theory describing the blade can be made at different levels of complexity depending on its use [6]. An example of a more complex version is the BECAS model (BEam Cross-section Analysis Software) (see, e.g., [7]). A simplified 1D momentum theory (see, e.g., [6]), the maximum aerodynamic load on a blade in the flap-wise direction can be simplified as a linear varying distributed load with vanishing load intensity at the root to a load intensity of:

(1) $$q_{L}=\frac{8}{27}\rho_{air}u_{R}^{2}\pi L$$

at the blade tip. Here, L is the blade length, $\rho_{air}=1.2\;{\mathrm{kg}/m}^{3}$ is the density of the air, and $u_{R}$ is the so-called rated wind speed for the turbine which corresponds to the wind speed when the turbine production is reaching the generator size. In the edge-wise direction, the load has, in [6], been assumed to be constant.

**Figure 1:**
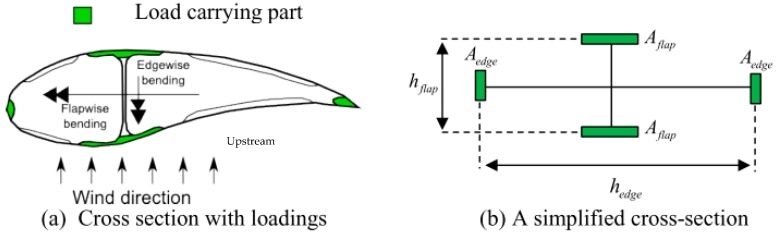
The simplified wind turbine blade cross-section used in the study.

Using the simplified cross-section shown in Figure 1, the flap and edge-wise moment in the blade (see, e.g., [5]) can be found as follows:

(2) $$M_{x}^{flap}=q_{x}^{flap}(L-x)\frac{L-x}{2}+\frac{q_{L}^{flap}-q_{x}^{flap}}{2}(L-x)\frac{2(L-x)}{3}$$

(3) $$M_{x}^{edge}=\rho_{blade}A_{blade}g(L-x)\frac{L-x}{2}$$

where, after substitution of the load intensity, can be written in stresses as:

(4) $$\sigma_{x}^{flap}=\frac{8}{81}\frac{\rho_{air}u_{R}^{2}\pi L^{3}}{A_{flap}h_{x}^{flap}}\left(1+\frac{x}{2L}\right){\left(1-\frac{x}{L}\right)}^{2}$$

(5) $$\sigma_{x}^{edge}=\frac{1}{2}\frac{\rho_{blade}A_{blade}gL^{2}}{A_{edge}h_{x}^{edge}}{\left(1-\frac{x}{L}\right)}^{2}$$

where $A_{blade}=2(A_{flap}+A_{edge})$. In order to achieve a constant stress level, $\sigma_{x}^{flap}=\sigma_{x}^{edge}=\sigma_{0}$ in the blade material, it can be seen from Equations (4) and (5) that the blade height, $h_{x}^{flap}$, and blade width, $h_{x}^{edge}$, should vary in the following manner:

(6) $$\frac{h_{x}^{flap}}{h_{0}^{flap}}=\left(1+\frac{x}{2L}\right){\left(1-\frac{x}{L}\right)}^{2}\;;\;\frac{h_{x}^{edge}}{h_{0}^{edge}}={\left(1-\frac{x}{L}\right)}^{2}$$

### 2.2. Stresses in a Wind Blade

**Figure 2:**
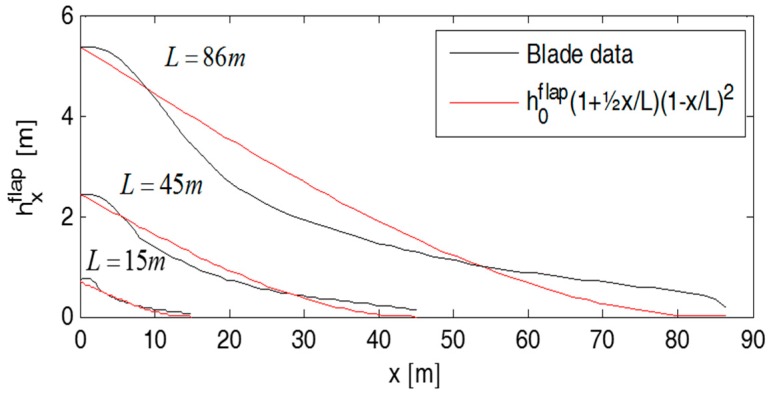
Typical blade geometries compared with the strength-determined geometries defined in Equation (6).

Following Equation (6) will result in blade geometries which are solely based on material strength. In reality, the blade geometry is given by a combination of structural and aerodynamic requirements. In Figure 2, Equation (6) is compared with typical blade cross-section variations, where the case L=86m, while the other two cases are representative blade geometries averaged from a number of known blade geometries. The blade height $h_{0}^{flap}$ and width $h_{0}^{edge}$ are specified such that Equation (6) is crossing the representative blade geometry in the largest value points which, for the thickness direction, is at the blade root while, for the width, it is at a point located away from that (see Figure 2). During this, it turns out that $h_{0}^{flap}$ and $h_{0}^{edge}$, as a good approximation, can be assumed to follow linear functions, with L as the blade length:

(7) $$h_{0}^{flap}=0.066L-0.369m\;;\;h_{0}^{edge}=0.130L+0.082m$$

Therefore, based on the parameters given in [6], the mass of a specific wind turbine blade can be estimated. Keeping in mind the assumption of a constant cross-section along the blade, the height and width variation are obtained aiming for a constant stress value throughout the blade. In addition to a blade design given by the material strength, the blade should also be stiff enough in order to avoid the blade hitting the tower. In [8], the distance from the blade tip to the tower is given as 18 m; this is, therefore, the maximum allowable deflection $w_{\max}=18m$ for a L=86m turbine blade. Solving the Bernoulli-Euler equations numerically, one obtains the rotation and deflection at the root section:

(8) $$\begin{array}{ccc} \theta_{i}=\theta_{i-1}+\frac{1}{2}\left(\frac{M_{i}}{EI_{i}}+\frac{M_{i-1}}{EI_{i-1}}\right)\left(x_{i}-x_{i-1}\right)\; & ; & i=1,\ldots n\\ \;w_{i}=w_{i-1}+\frac{\theta_{i}+\theta_{i-1}}{2}\left(x_{i}-x_{i-1}\right)\; & ; & i=1,\ldots n \end{array}$$

where w is the transverse deflection, $M_{x}$ is the moment in the beam at location x, and E is the material stiffness, $\frac{d^{2}w}{\partial x^{2}}=\frac{M_{x}}{EI_{x}}\;\mathrm{with}\;I_{x}=\frac{1}{2}A^{flap}{\left(h_{x}^{flap}\right)}^{2}$.

## 3. Three Dimensional Modelling of Composite Degradation under Tensile Loading

An important task of the analysis of wind blade materials is the prediction of service properties and lifetime of the wind blades. This information can be obtained from computational modelling, which can be carried out at several scale levels: mesolevel (laminates, sandwiches of the blade shells, fiber bundles), microlevel (fibers and matrix), and nanolevel (interfaces, nanoparticles). In order to simulate the deformation and strength of fiber reinforced composites, a number of approaches can be used, e.g., analytical models (often based on the shear lag model [9], among these models, one can mention the break influence superposition (BIS) technique [10], Green function model [11,12], etc.); the fiber bundle model (FBM) developed initially by Daniels [13], and further improved and generalized by many groups (see, for example, [14]), fracture mechanics-based models (often applied to the cases of brittle matrix and fiber bridging [15,16,17]), continuum damage mechanics-based models [18,19], and numerical continuum mechanical (often, finite element-based) models [20,21,22,23,24,25,26,27], and multiscale models [28]. A detailed overview of models of fiber-reinforced composites can be found elsewhere [25,26].

Below, the micromechanical unit cell models of composites at the level of the fiber/matrix and bundles are reviewed.

### 3.1. Computational Modelling of Micromechanisms of Degradation of Wind Blade Composites

In order to analyze the micromechanisms of damage in wind energy composites, micromechanical finite element simulations have been used [29]. A series of programs has been developed, which should automate the steps of the generation of 3D microstructural models of composites. After a 3D microstructural model of a material with a complex microstructure is generated, the numerical testing of the microstructure is carried out with the use of commercial finite element software, Abaqus 6.13 (Dassault Systèmes, Vélizy-Villacoublay, France). In order to model the fiber cracking, we introduced potential fracture planes (in theform of damageable cohesive elements) in random sections of fibers, suggested by González and LLorca [30]. The random arrangement of the potential failure planes in this case reflects the statistical variability of the fiber properties. A similar concept was used to simulate the interface cracking of composites. Given that surfaces of fibers can be rather rough, and the interface regions in many composites contain interphases, the interface debonding was considered not as a two-dimensional opening of two contacting plane surfaces, but rather as a three-dimensional process in a thin layer. Thus, the interface was represented as a “third (interphase) material layer” between the homogeneous fiber and matrix materials. The thickness of the interface layer can be varied in simulations. The damage evolution in the damageable layers, placed in random sections of fibers, as well as in the matrix and interphase layers, was modeled using the finite element weakening method. The idea of this approach is that the stiffness of finite elements is reduced if a stress or a damage parameter in the element or a nodal point exceeds some critical level. This approach has been realized in the Abaqus subroutine User Defined Field. In this subroutine, the phase to which a given finite element in the model is assigned is defined through the field variable of the element.

**Figure 3:**
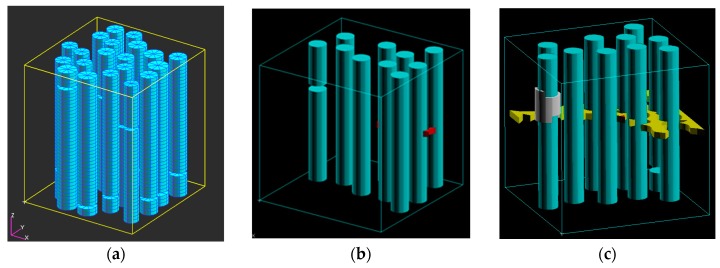
3D FE micromechanical model: (**a**) example of a 3D unit cell; (**b**) competition of damage modes: one failed fiber and a few microcracks in the matrix (red); and (**c**) two fibers have failed, the interface crack is formed in the vicinity of a fiber crack and the matrix crack is formed (ε = 0.015) (reprinted from [29] with kind permission from Elsevier).

Figure 3a shows an example of the generated FE models with 20 fibers, and removed layers of potential fracturing. A number of three-dimensional multifiber unit cells have been generated automatically with the use of these programs and were subject to a uniaxial tensile displacement loading along the axis of the fibers. As output results, the stress-strain curves and the damage strain curves were obtained, as well as the stress and strain, and damaged element distributions in the unit cells (see [29]). On the basis of the simulations, it was concluded that homogeneous fibers ensure a higher strength of a composite at the pre-critical load. However, the fibers with randomly-distributed strengths lead to the higher strength at post-critical loads, i.e., after the fiber cracking started.

Let us consider now the interaction between all three damage modes in composites (matrix cracks, interface damage, and fiber fracture). Figure 3b,c show the results of simulations in the unit cell with 20 fibers: damage formation in the fibers, interface and matrix. The damage evolution begins by formation of a crack in a fiber and (in another, rather far site) in the matrix (ε = 0.01). Then, the interface crack forms near the fiber crack, and the large matrix crack is formed (ε = 0.015).

It is of interest that, in the case when all the three damage mechanisms are possible, the competition between the matrix cracking and the interface debonding is observed. In the area where the interface is damaged, no matrix crack forms; vice versa, in the area where the long matrix cracks are formed, the fiber cracking does not lead to the interface damage. One can conclude that local weak places in composite interfaces can be rather beneficial for the composite strength and toughness: they can prevent the matrix failure (by channeling the fracture energy into interface defects), and even delay the fiber failure. Practically, it means that a heterogeneous interface (interface with both weak and strong regions) can prevent the matrix failure and, therefore, ensure the integrity of the material.

### 3.2. Fiber Bundle Modelling with an Experimentally-Determined Fiber Distribution

**Figure 4:**
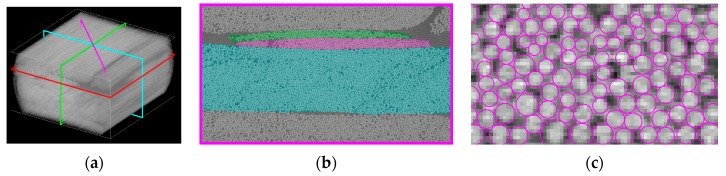
Segmentation of 0°, magenta: 45°, and green: 90° bundles, where the 0° is in the loading direction. (**a**) Cut planes, (**b**) Bundle segmentation, (**c**) Fibre segmentation.

The model of the Section 3.1 above was applied to an ideal unidirectional structure. In order to simulate real structures of uni-directional non-crimp fabric-reinforced epoxy composites, an approach based on 3D X-ray tomography scanning is used [31]. The bundle and fiber/matrix structure of the composite is segmented as follows: A complete 0° load-carrying bundle, as well as complete 90° and 45° bundles, are contained in the 3 mm × 3 mm × 3 mm FoV scan. This makes it possible to choose three cut planes. Each cut plane is orthogonal to one of the bundles and contains its complete cross-section, as shown in Figure 4a. All subsequent analysis is performed in those three 2D images, separately for each of the fiber bundles. The bundles are manually segmented, and the example of a 45° bundle is shown in Figure 4. For detecting the individual fibers, the automatic approach of Emerson et al. [32] is applied for estimating fiber centers. Fibers are then modeled as circles with the diameters calculated so that no fibers overlap, but all fibers touch at least one neighboring fiber. An example of the segmentation results for the fibers from a 45° bundle is shown in Figure 3, right.

Compared with the expected fiber diameter of 17 µm and 16 µm for the load carrying 0° and the backing bundles (45°, 90°), the segmentation is found to underestimate the diameters somehow. As it can be seen in the zoomed image in Figure 4, right, it is difficult to distinguish individual fibers, so some uncertainty may also be expected for the chosen resolution. The current segmentation is performed for 2D cross-sections and a full 3D based segmentation is expected to give an improved precision. Additionally, a scan with a higher zoom and a better resolution would improve the precision significantly. The last column in Table 1 shows the estimated tex-values based on the segmentated data. Compared with the tex-values reported for the fabric, the 0° load-carrying bundle is found to be overestimated, while the backing bundles are found to be underestimated. Based on the under-estimation of the fiber diameters, an under-estimation of the tex-values was expected for all the bundles. Nevertheless, the boundary between the load carrying 0° bundles can be hard to identify on a 2D slice, so the segmented results may include some part of a neighboring bundle.

**Figure 5:**
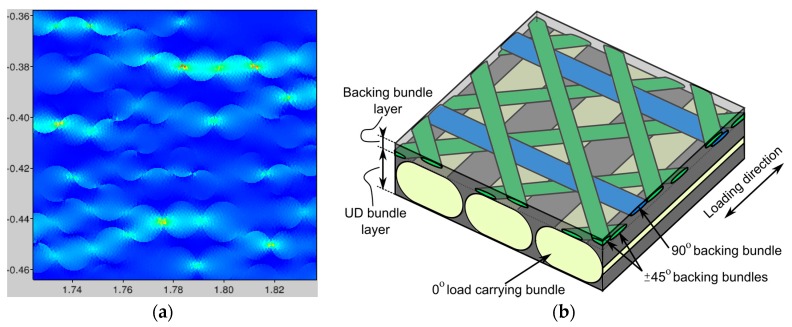
(**a**)The normal horizontal stress contour plot of a transverse-loaded 45° backing bundle and (**b**) the 3D structure where the resulting constitutive law will be implemented.

Based on the segmented fiber architecture, a two-dimensional micromechanical model oriented orthogonally to the bundle orientation is built (see Figure 5a). In practice, the fiber architecture is segmented as fiber center points and diameters. Based on this, a representative rectangular part of the bundle area is selected for the finite element model and subsequently loaded in the transverse direction. As the much stiffer fibers will constrain the matrix deformation in the fiber direction, a plane strain linear triangle element is used. For the 45° bundle, a 1.0 × 1.0 mm representative box with 443 fibers is selected. Only the solution for a very small part of the simulated representative box is shown in Figure 5a. The local fiber volume fraction inside the representative volume is found to be $V_{f}=0.56$. Based on the micromechanical finite element model, the transverse stiffness and Poisson’s ratio are found to be $E_{FEM}=13.8\;\mathrm{GPa}$ and $\nu_{FEM}=0.49$, whereas the inverse rules of mixture will give $E_{InvRoM}=6.4\;\mathrm{GPa}$ and the Halpin-Tsai estimate will give $E_{Halpin-Tsai}=11.8\;\mathrm{GPa}$. When the full 3D constitutive relation of the bundle structure is obtained, this will be applied on the segmented bundle structure, which is sketched in Figure 5.

## 4. Compressive Strength of Wind Turbine Composites

The downwind side of the blade and the spar of the blade are subject to compressive loading. The failure mechanisms of unidirectional composites under compressive loading differ strongly from those under tensile loading [33]. The compressive strength of composites is often sufficiently lower than their tensile strength, especially for carbon fiber composites where it can be 50–60% of the tensile strength.

In this section, two approaches to simulate the compressive strength of composites are considered: a statistical, fiber bundle-based model [34], and a smeared-out compressive approach [34]. The first analytical approach can be easily extended to more complex loading (fatigue, complex structures), while the smeared out model is applicable for larger amounts of fibers.

### 4.1. Statistical Model of Compressive Damage Evolution

**Figure 6:**
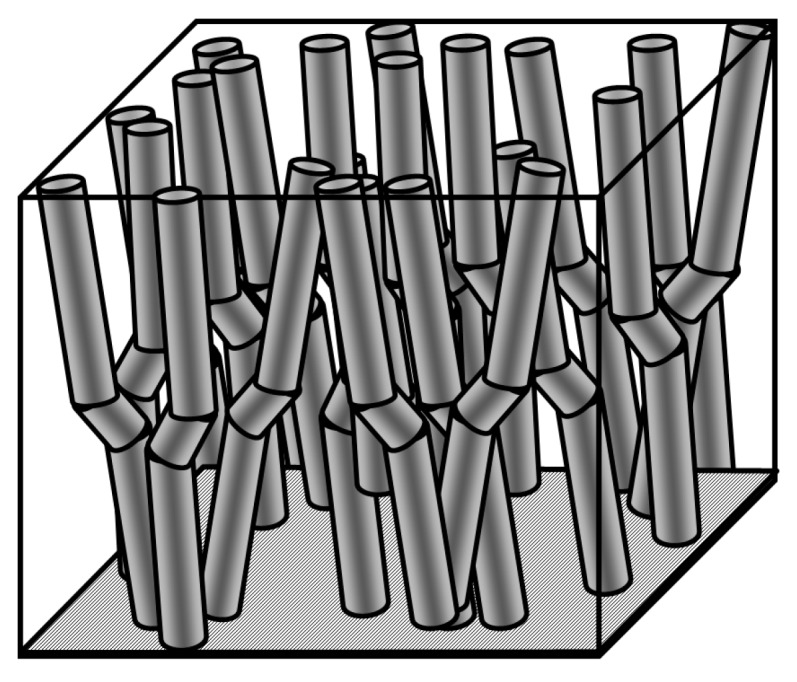
A schema of the multifiber unit cell with random misalignments and fiber misalignments.

In this section, we investigate the effect of microstructures and the statistical distribution of microstructural parameters of the composites on the damage, and the compressive and fatigue strength of carbon fiber-reinforced composites under compressive and cyclic loading. A computational model of a composite with a number of randomly-distributed and randomly-misaligned fibers has been developed [34]. The model is based on the Monte-Carlo method and the Budiansky-Fleck fiber kinking condition. The fibers are randomly arranged in the cell, using the RSA (random sequential absorption) algorithm. The misalignment angles are assigned to each fiber, using random normal number generator (with truncated Gaussian probability distribution, from −3° to 3°). Then, the unit cells were subject to axial loading (or repeated axial loadings). For each fiber, the kinking condition is checked according to the Budiansky-Fleck formula. If one or several fibers kink, the stress is redistributed over the remaining fibers, thus, increasing the load on the remaining fibers and the likelihood of their kinking. The load sharing on the remaining fibers after kinking of some fibers was calculated using the “effective stress concept” of damage mechanics and the power load sharing law. The schema of the multifiber unit cell with random misalignments is given in Figure 6.

Two scenarios of the load redistribution after the fiber kinking were considered, depending on the “rate” of the load redistribution: “quick” loading (when the fibers are loaded, and fail independently, and the load redistribution takes place only at the next loading), and “slow” loading (after a fiber is failed, the stress on the remaining fibers increases instantly according to the “effective stress concept” and “load sharing rule”, and so on for all the fibers which fail successively one after another). In the first case, a *j*-th fiber does not “know” that the *i*-th fiber failed, until the next cycle of loading (the “quick” loading term reflect non-sharing of the load, as if it was quicker than the load transfer from failed to intact fibers). Only in the next cycle, the load is redistributed over remaining fibers. In the latter case, the fibers kink one after another, depending on the misalignment of each fiber. Thus, the “slow” loading leads to autocatalytic fiber kinking, caused not by the increase of the applied load, but rather by the load redistribution after the fiber begins to kink.

**Figure 7:**
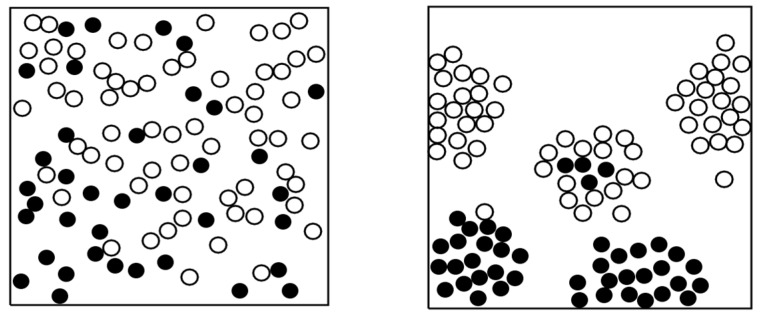
Distribution of failed fibers in the case of clustered and random homogeneous fiber arrangements. The case of 100 fibers, five clusters, vc = 20%, damage parameter of 0.37 in random structures, and 0.43 in clustered structures (reprinted from [34] with kind permission from Elsevier).

This model can be applied, among others, to analyze the effect of the fiber clustering on the compressive strength of the composites. In order to make the clustering effect better visible, we considered the composite with 20% volume content of fibers. The unit cell models with random and clustered arrangements of fibers were subject to loadings (“quick” loading scheme). In the simulations, it was observed that the fiber clustering has no effect on the damage at the first “quick” loading. However, at the second “quick” loading, the composites with clustered fiber arrangements demonstrated sufficiently higher damage. Figure 7 shows the distributions of failed fibers in the cases of clustered and random homogeneous fiber arrangements, for the N = 100 (five clusters), after the second loading cycle. One can see that the clustered fiber arrangement leads to the quicker failure of the composite, due to the effect of the load redistribution. While there might be no difference between the clustered and homogeneous fiber arrangement if the material is not pre-damaged, the clustered arrangement leads to the much quicker failure of fibers at the second loading (or if the material is pre-damaged). For instance, at the compressive stress is 1500 MPa, the damage in the composite with clustered fibers is 32.5% higher than in the composite with homogeneously-arranged fibers.

### 4.2. Effect of Local Fiber Misalignment and Wrinkles on Compression Strength of the Composite

**Figure 8:**
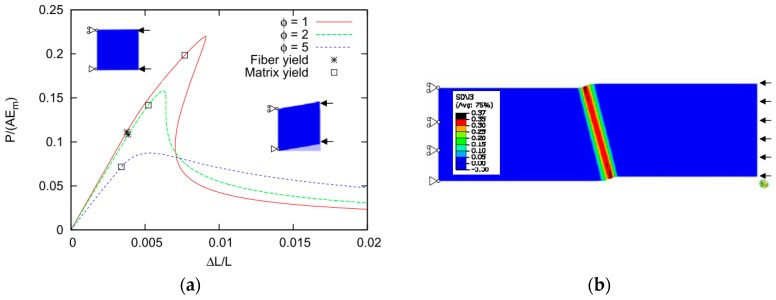
Smeared out finite element modeling of the fibre reinforced polymer matrix composite. (**a**) Smeared out material behavior; (**b**) Kink-band formation with yielding zone.

As an alternative to modelling the individual fibers, which is far too detailed an approach when we are talking about wind turbine blades which consist of millions of fibers, a smeared out approach can be used where both the fiber and matrix constituents can be modelled as a non-linear elastic-plastic model, but without modelling them individually. Such an approach is used in [35] and the example on predictions for a specific material combination is shown in Figure 8. It has been found that the kink-band formation is highly dependent on the plasticity of the matrix material (the soft phase) in interaction with the fiber misalignment. Therefore, it has, e.g., been demonstrated that temperature change from 20 °C to 50 °C can easily reduce the compression strength by 30%. In [36], the individual fiber orientation was segmented in a filament-wound carbon fiber and a glass fiber composite. Thereby, rather good agreement between experimental measurement and numerical predictions were obtained.

Smeared out models are not limited to modelling the compression failure at the fiber matrix level. In [35], a 2D FE model of alternating layers of elastic-plastic matrix and elastic orthotropic fiber laminae was modelled. Additionally, at this larger scale, the compression strength of a wrinkled laminate was found to be strongly dependent on the maximum initial lamina misalignment angle and the plasticity of the matrix material. Finally, it is found that in hybrid wrinkles, an increasing amount of carbon increases the compression strength.

In addition to through-thickness wrinkles we considered partial wrinkles [37]. It was shown that the undisturbed laminate next to the wrinkle along the length reduces the kinking strength which, afterwards, increases when the undisturbed laminate is on top of that added below and/or above the waviness. The delamination study demonstrates that the global wrinkle model actually fails by delamination for an initial misorientation equal to, or greater than, 4°.

## 5. Computational Modelling of Hybrid and Hierarchical Composites

Recently, several new promising directions of wind blade composites development has attracted the attention of researchers, namely, hybrid and nanoengineered materials.

In a number of works, the strength and damage mechanisms of hybrid composites were studied [38,39,40,41,42,43]. It was shown that the incorporation of glass fibers in carbon fiber-reinforced composites allows the improvement of their impact properties and tensile strain to failure of the carbon fibers. For the analysis of nanocomposites, a number of different micromechanical models have been developed; for instance, approaches based on composite models (Halpin-Tsai model, Mori–Tanaka theory, Eshelby model, and shear lag models [44,45,46]) and molecular mechanics/molecular dynamics [47,48,49,50,51,52]. A number of special nanostructure-based models were developed, e.g., the effective particle model [53], dilute suspension of clusters model [54], effective clay platelet (effective nanofiller) [55], effective cluster material [56], etc. In this section, the computational finite element of composites, developed in Section 3 and Section 4, are applied to the analysis of the hybrid and hierarchical composites.

### 5.1. Modellling of Hybrid Composites

**Figure 9:**
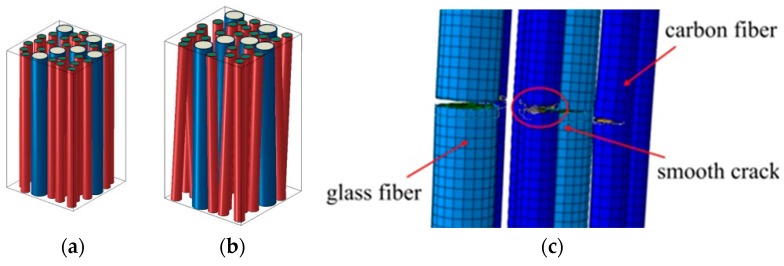
Examples of unit cell FE models of glass/fiber hybrid composites (**a**) aligned; and (**b**) misaligned fibers, and simulated cracking in fibers (**c**) (reprinted from [57] with kind permission from Elsevier).

While the effect of the hybrid structures of unidirectional composites on the stiffness and weight is apparent, and can be well described by the rule-of-mixture (higher fraction of lightweight and stiff fibers means a proportional increase of the composite stiffness and reduction of the weight), the effect of the hybridization on the damage and fatigue resistance is more complex. For the analysis of structure-strength/damage resistance relationships of hybrid composites, computational experiments have been carried out with the use of the probabilistic fiber bundle model and micromechanical multifiber FE unit cell models [33,57,58,59]. Figure 9a,b show examples of the unit cell FE models of glass/fiber hybrid composites, with fiber misalignment. Figure 9c shows the simulated cracking in fibers.

**Figure 10:**
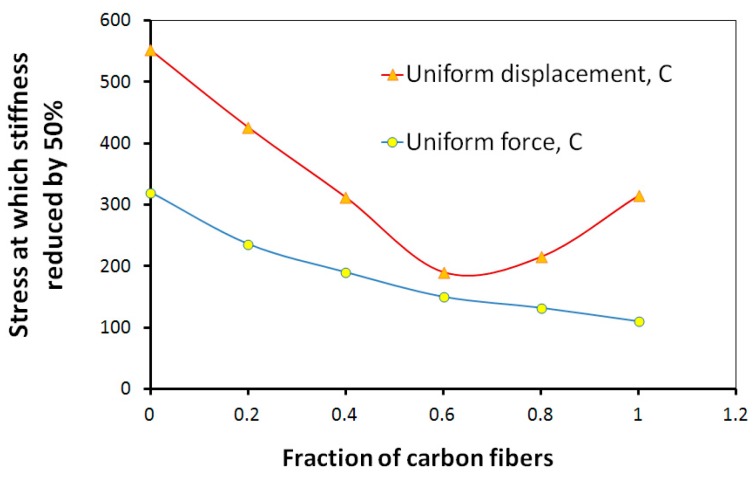
Critical stress (corresponding to D = 50%) plotted versus the fraction of carbon fibers in hybrid glass/carbon fiber composites (from [57], with kind permission of Elsevier).

Further, the statistical model of composite cracking described in the previous section was applied to the hybrid glass and hybrid composites. Figure 10 shows the critical stress (corresponding to the load at which 50% of fibers fail), plotted versus the fraction of carbon fibers in hybrid glass/carbon fiber composites [57], obtained using this model.

One can see that the strength of hybrid composite decreases when the fibers are mixed, reaches lowest point at the fraction of carbon fibers 40–50% (of all fibers) and then begins to grow sharply, to achieve the highest point for the pure carbon composite. Similar results were obtained experimentally [60].

### 5.2. Nanoengineered Composites

It has been demonstrated [61,62,63,64,65,66,67] that nanoparticles distributed in the polymer matrix or at the fiber/matrix interface of the composites should enhance the compressive strength and fatigue lifetime of the composites. In order to analyze the effect of the secondary nanoparticles on the mechanical behavior and strength of composites, computational multiscale models were developed, which include the fiber/matrix interaction at the higher-scale level (microlevel) and nanoparticles/epoxy matrix interaction at the nanolevel.

**Figure 11:**
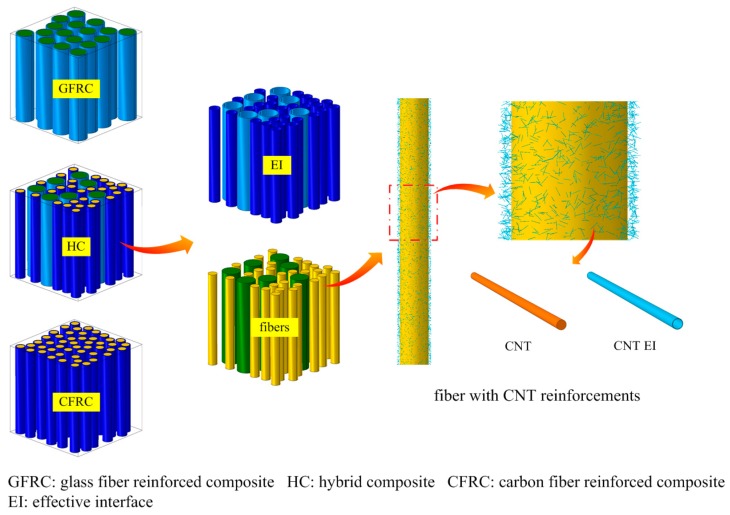
Example of a unit cell model of CNT-reinforced hybrid composite. Reprinted from [62] with kind permission of Elsevier.

Figure 11 shows a schema of a unit cell model. In this model, polymer composites with glass fibers (GFRC), carbon fibers (CFRC), and hybrid fibers (HC) are reinforced with carbon nanotubes (CNT). The aspect ratio of CNTs was 1000, and they were distributed in the sizing of the fibers. The volume fraction of the CNT reinforcement is 0.46%. As a result of simulations, it was shown that the CNT enhances the fatigue performance (maximum stress and lifetime) in all considered composites. For the very low cycle loading, the CNT reinforcement leads to 25–43% increase in the stress while, for the millions of cycles, the CNT effect increases the stress by 64–120%. Similarly, the nanoclay particles located in the matrix or in the fiber sizing, ensure drastically enhanced fatigue lifetimes of composites [62].

**Figure 12:**
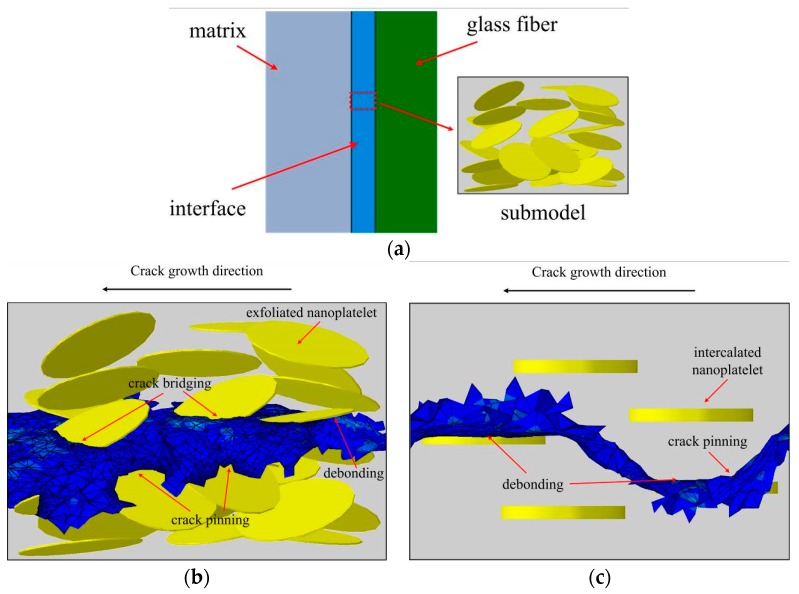
Schema of the model of thehierarchical fiber composite with nanoclay reinforcements (**a**) and simulated crack paths in submodels (aligned and randomly-oriented platelets (**b**,**c**)). Reprinted from [66] with kind permission of Elsevier.

Figure 12a shows a schema of the model of the hierarchical fiber composite with nanoclay reinforcements (a) and simulated crack paths in in-between platelets observed in the simulations (aligned and randomly-oriented platelets, b and c) [66]. The composites with nanoclay reinforcement achieve the same fatigue life (taken exemplarily at 5.68 × 10^7^ cycles) as neat composites, while subject to 2–3.5 times higher loadings. Composites with the nanoplatelets localized in the fiber/matrix interface layer (fiber sizing) ensure much higher fatigue lifetimes than those with the nanoplatelets in the matrix: 43–49% higher applied stress corresponding to the selected lifetime of 5.68 × 10^7^ cycles.

In further simulations, composites with nanoclay secondary reinforcements were analyzed [62]. Figure 12b,c show the simulated crack path in the interface area of the composite with aligned and randomly oriented nanoclay particles. As a result it was shown that the nanoclay (NC) secondary reinforcement allows to increase fatigue resistance drastically. Composites with NC achieve the same fatigue life (taken exemplarily at 5.68 × 10^7^ cycles) as neat composites, while subject to 2–3.5 times higher loadings. Composites with NC in fiber sizing, with exfoliated NC, and aligned NC ensure much higher fatigue lifetimes than with NC in the matrix, with clustered or randomly-oriented NC (respectively).

## 6. Conclusions

An overview of computational studies of composites for wind energy applications carried over from recent years in the Composite and Materials Mechanics Section, DTU Wind, is presented. In this review, the computational models of the deformation and damage evolution of various composites are presented. In the numerical experiments, damage mechanisms were analyzed. The competition between matrix cracking and interface debonding was observed in numerical experiments. In the area, where the interface is damaged, no matrix cracks form, and vice versa, in the area where the long matrix cracks are formed, the fiber cracking does not lead to interface damage. One can conclude that local weak places in composite interfaces can be rather beneficial for the composite strength and toughness: they can prevent matrix failure (by channeling the fracture energy into interface defects), and even delay the fiber failure. Practically, this means that a heterogeneous interface (interface with both weak and strong regions) can prevent matrix failure and, therefore, ensure the integrity of the material. In the statistical model of composite compression, it was observed that the clustered, bundled fiber arrangement leads to the quicker failure of the composite due to the effect of the load redistribution. While there might be no difference between the clustered and homogeneous fiber arrangement if the material is not pre-damaged, the clustered arrangement leads to the much quicker failure of fibers at the second loading (or if the material is pre-damaged).

Compression strength of a wrinkled laminate was found to be strongly dependent on the maximum initial lamina misalignment angle and the plasticity of the matrix material. Finally, it is found that, in hybrid wrinkles, an increasing amount of carbon increases the compression strength. The undisturbed laminate next to the wrinkle along the length reduces the kinking strength, which afterwards increases when undisturbed laminate are on top of that added below and/or above the waviness.

Further, the effect of hybrid and nanoengineered structures on the performance of the composite was studied in computational experiments. It was shown that the strength of the hybrid composite decreases when the carbon fibers replace glass fibers, reaching the lowest point at a fraction of carbon fibers of 40–50% (of all fibers) and then begins to grow sharply, to achieve the highest point for pure carbon composite. Further, it was observed that the nanoscale reinforcement enhances the fatigue performance (maximum stress and lifetime) in all considered composites. For very low cycle loading, the nanoreinforcement leads to a 25–43% increase in the stress while, for the millions of cycles, the CNT effect increases the stress by 64–120%. Similarly, the nanoclay particles located in the matrix or in the fiber sizing ensure drastically enhanced fatigue lifetimes of the composites.

## Figures and Tables

**Table 1:** Quantification from the fiber segmentation inside the bundles.

Bundle	Number of Fibers	Average Fiber Diameter	Total Bundle Area	Total Fiber Area	Local Fiber Volume Fraction	Estimate Tex-Value
0°	5954	15.8 µm	1.926 mm^2^	1.197 mm^2^	0.62	3113
45°	794	14.9 µm	0.245 mm^2^	0.142 mm^2^	0.58	369
90°	375	15.4 µm	0.125 mm^2^	0.071 mm^2^	0.57	185
